# Urban transmission of mosquito-borne flaviviruses – a review of the risk for humans in Vietnam

**DOI:** 10.1080/20008686.2019.1660129

**Published:** 2019-08-30

**Authors:** Thang Nguyen-Tien, Åke Lundkvist, Johanna Lindahl

**Affiliations:** a Department of Medical Biochemistry and Microbiology, Uppsala University, Uppsala, Sweden; b International Livestock Research Institute, Hanoi, Vietnam; c Department of Clinical Sciences, Swedish University of Agricultural Sciences, Uppsala, Sweden

**Keywords:** Mosquito-borne flavivirus, Dengue virus, Zika virus, Japanese encephalitis virus, urban transmission, Vietnam

## Abstract

Vietnam is a tropical country where mosquito-borne diseases are common. This review explores the transmission of mosquito-borne flaviviruses in urban areas of Vietnam. It concludes that urban transmission has mainly been studied for Dengue virus, and so far, much less for Japanese encephalitis virus. Dengue is the most common flavivirus in Vietnam. Due to fast urbanization and favorable climatic conditions, the viral transmission concentrates mainly to large cities with high population density including Ha Noi, Nha Trang and Ho Chi Minh. Human cases of Japanese encephalitis have been controlled by an expanded immunization program. However, this virus is still circulating throughout the country, also in cities due to the pig rearing practices in urban and peri-urban areas. Zika virus is an additional major concern because it has long circulated in the Northern area and is now increasingly diagnosed in urban areas of the Central, Central Highlands and Southern regions using the same mosquito vectors as Dengue virus. There was alarge outbreak of Zika disease from 2016 to early 2017, with most infections observed in Ho Chi Minh city, the largest town in Vietnam. Other flaviviruses circulate in Vietnam but have not been investigated in terms of urban transmission.

## Introduction

The Flavivirus genus comprises more than 70 different viruses belonging to Flaviviridae family, including the five well-known mosquito-borne viruses Dengue (DENV), Japanese encephalitis (JEV), Zika (ZIKV), West Nile (WNV) and Yellow fever (YFV) viruses [1,2]. These are all common viruses causing life-threatening infectious diseases in humans via mosquitoes. According to the World Health Organization (WHO), flaviviruses transmitted by mosquitoes cause millions of human cases and nearly a million of human deaths every year [3]. With increased urbanization and significant climatic changes, it is likely that we will see increased disease transmissions in cities, and additional mosquito-borne viruses may adapt to these circumstances [4]. Humans are dead-end hosts for JEV infections [5,6] and WNV transmission [1,7], but are the major amplifying hosts in the transmission cycle of DENV [2] and ZIKV, and involved in the transmission cycle of YFV [1,8]. Therefore, it should be noted that all these flaviviruses pose a major threat to the human society.

**Figure 1.**
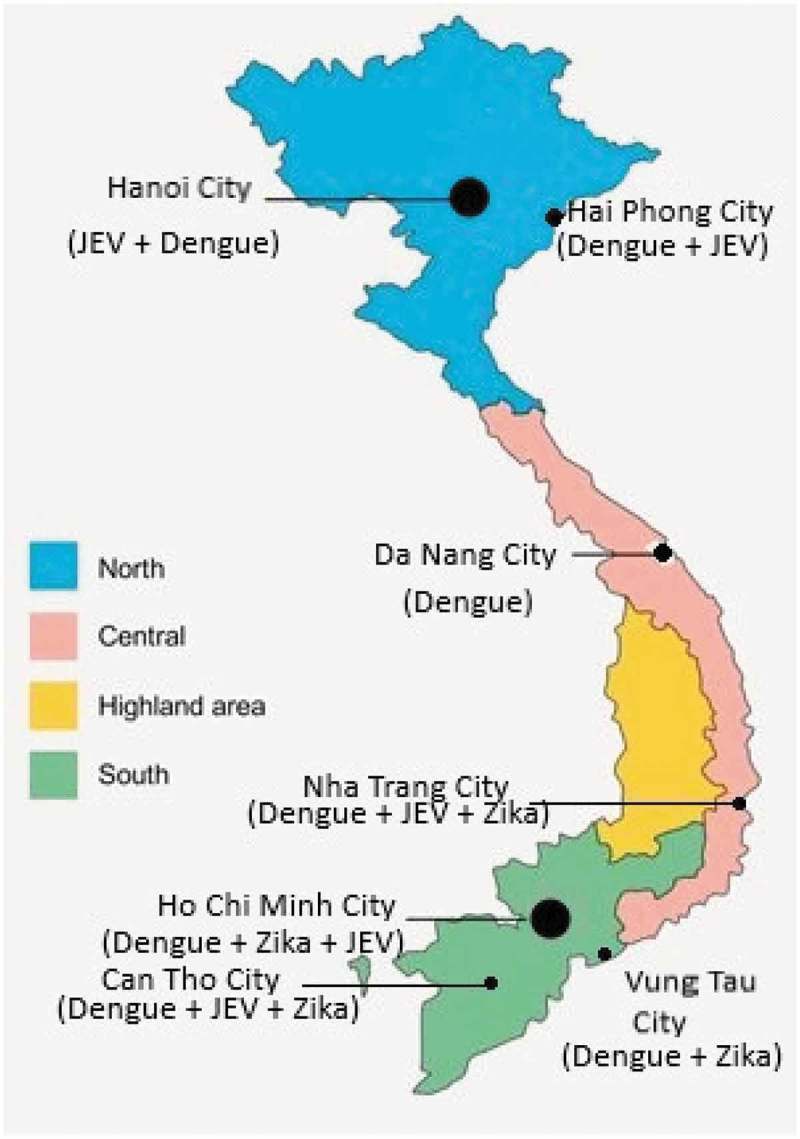
Distribution of three flaviviruses in big cities in Vietnam.

Vietnam is a tropical country in Southeast Asia where three of these mosquito-borne viruses (JEV, DENV and ZIKV) are endemic. Geographically, Vietnam can be divided into four regions including the Northern part, the Central Part, the Highlands Central part and the Southern part (Figure 1).

This review focuses on the transmission of flaviviruses in urban areas of those regions, where the increasing urbanization contributes to an increased risk for mosquito-borne diseases.

### Japanese encephalitis virus

Japanese encephalitis virus is prevalent in 24 Asian countries, encompassing almost half of the world’s population [9–11]. This flavivirus is the most common mosquito-borne pathogen causing viral encephalitis [1,7] and has been estimated to cause 68,000 human cases each year in global [9,10,12]. About 75% of all Japanese encephalitis (JE) cases are observed among children in the age group 0–14 years [12]. The clinical symptoms of a JEV infection are not always evident, and some cases develop only mild fever and headache. In case of severe symptoms such as high fever, headache, neck stiffness, disorientation, coma, seizures, spastic paralysis, the fatality rate can range from 20% to 30%, while 30–50% of the survivors will suffer severe neurologic or psychiatric sequelae [10,12].

The enzootic transmission cycle of JEV involves mosquitoes, pigs and water birds [10]. Mosquitoes of the genus *Culex*, and in particular *Culex tritaeniorhynchus*, were early described as the most important vectors of JEV [13]. JEV has been isolated from more than 30 species of mosquitoes since it was first isolated in the 1930s [5], but mosquitoes from the *Cx. vishnui* subgroup, especially *Cx. tritaeniorhynchus* are still considered the most important vectors [1,4]. *Cx. gelidus* and *Cx. fuscocephala* are also recognized vectors, and the anthropophilic *Cx. quinquefasciatus* has been shown to be naturally infected and capable of transmitting JEV experimentally [14,15]. In addition, mosquitoes from other genera, such as *Aedes, Armigeres, Anopheles* and *Mansonia* have been shown to carry the virus or to be able to transmit it experimentally [6,14,16–18]. However, it is not yet known if these mosquitoes have any significant role in the epidemiology of JEV. Birds and pigs serve as reservoirs and amplifiers of the virus, whereas humans and other animals are considered accidental dead-end hosts [4,11]. Similar to human infections, JEV may cause fatal encephalitis in horses, whereas pigs often are only sub-clinically infected, or show reproductive symptoms, such as abortion or stillbirths [19]. Many other domestic animals seroconvert without clinical symptoms, and dogs have recently been suggested as possible sentinels. Serological screening of dogs may be more representative for assessing human risks in urban environments, compared to screening of pigs [20]. Emergence of JEV can be associated with both the growth of the human population and intensification and structural changes in agricultural practices. In Asia, the agricultural sector is rapidly growing. Especially the closeness to pigs and rice fields are established factors that increase the risk of contracting JE, and rice fields are favoured breeding sites for *Cx. tritaeniorhynchus* and other species in the *Cx. vishnui* subgroup [21–23], and also *Cx. gelidus* [24], but this species is also known to breed in wastewaters [25]. Therefore, the disease is most common in rural areas where growing rice fields and irrigation systems make up breeding grounds for vectors [11]. In contrast, the urban *Cx. quinquefasciatus* often breeds in dirty stagnant waters, sewers and drains [26]. In an outbreak of JEV in Australia; closeness of stagnant water and the density of the human population was believed to be important risk factors for infection [27]. However, the epidemiology of JEV is likely to change with ongoing demographic and climate changes. The effects that the climate changes may have on disease transmission are difficult to predict since both vector and host factors will be affected [28]. Although JE is generally viewed as a rural disease, JEV infection is also observed in pigs and humans living in urban environments [4,29–31].

In Vietnam, JEV was isolated for the first time in 1951 [32]. Until now, the incidence rate of JE in humans has diminished dramatically after introduction of an immunization program in the form of campaigns in high-risk areas in 1997. Until 2007, JE vaccination schemes covered 65% districts of Vietnam and was further scaled up nationwide in 2015 through an expanded program of vaccination [32,33]. Before 2003, JEV was endemic throughout the country with the annual incidence of 1000–3000 cases, mainly in rural areas [11,34]. However, the virus has also been circulating in cities and their vicinities for long [35], where JEV has been isolated from pigs, mosquitoes and humans [36].

Regarding the mosquito vector of JEV, in 2003, an entomological study was conducted in a suburban area, namely the Ha Tay province (at present part of Hanoi city since 2008). Maiko Hasegawa and co-workers found that the *Culex* population was predominant as compared to other mosquito species and its abundance was associated with the density of cattle [37]. Another survey in both urban and rural areas of eight cities/provinces throughout Vietnam between 2006 and 2008 indicated that JEV existed in adult mosquitoes collected at different time points of the year and in different provinces in three out of four regions of Vietnam. *Cx. tritaeniorhynchus* collected in the Ha Tay province in May 2006 were found positive for JEV [34]. Lindahl and co-workers investigated vectors of JEV in the urban environment of Can Tho city in southern Vietnam and found that vectors known to be competent transmitters of JEV were present in and around urban homes. Pigs in the close vicinity were associated with an increased number of competent vectors, in particular *Cx. tritaeniorhynchus*. An increased density of people in the household was related to higher numbers of *Cx. quinquefasciatus* [38]. *Cx. quinquefasciatus* is a mosquito species known to prefer urban habitats and breed in stagnant waters such as sewers and other polluted waters [26], explaining its abundance in the study, and this species was indicated as a potential urban vector of JEV in Can Tho city [39]. Additionally, a recent study revealed that 60.4% of pigs sampled in slaughterhouses in Hanoi city were infected with JEV [40]. It is possible that JE may become an increasing concern as increased urbanisation combined with a need to keep livestock (in particular pigs) creates new conditions for disease transmission in cities [38].

In humans, surveillance data from the five Northern provinces of Vietnam: Thai Binh, Hai Phong, Thanh Hoa, Hai Duong, Bac Giang in 2004–2005 showed that more than half of 421 samples were positive for IgM against JEV, with 91% of these JEV infections observed in children under 15 years. Remarkably, among these five provinces, Hai Phong which is an urban city, recorded 61% of acute encephalitis syndrome cases laboratory confirmed as JEV [32]. A recent serological survey of children between 1 and 10 years in Nha Trang city showed that JEV was still co-circulating with DENV, but at a lower rate. In 80 randomly selected ELISA test-positive samples; 21.3% were found seropositive for JEV; 87.5% were found positive to DENV, while 17.5% were positive to both JEV and DENV by a plaque reduction neutralization test [41].

### Dengue virus

DENV is one of the most important mosquito-borne viruses causing disease in humans throughout all tropical areas [42,43]. Every year, DENV infections result in 50–100 million cases of symptomatic illness in over 100 affected countries [44]. The total number of DENV infections has been estimated to 390 million with approximately 70% of the infections occurring in Asia [45]. More than half (3.97 billion people) of the world’s population is at risk of being infected with DENV [46]. There are two diverse transmission cycles that maintains DENV endemic: a human cycle and a sylvatic cycle. The human cycle includes the transmission between *Aedes* mosquitoes and humans, where humans acts as the only known reservoir. DENV is transmitted from humans to humans through the bites of female *Aedes* mosquitoes. In contrast, the sylvatic cycle involves non-human primates and *Aedes* mosquitoes [47]. There are four distinct serotypes of DENV named as DENV-1, DENV-2, DENV-3, and DENV-4 [48]. Recently, another type called DENV-5 and related to the sylvatic cycle has been described [49]. The serotypes 1–4 are all spread globally today. Infection with any DENV serotype can produce a spectrum of illness ranging from a mild, non-specific febrile syndrome, to classic dengue fever (DF), or more severe forms of disease, such as dengue haemorrhagic fever (DHF) and dengue shock syndrome (DSS), that can be fatal [48]. The *Aedes* vectors prefer clean water for breeding; therefore, this virus can also be transmitted in rural areas where clean water is accessible [50]. However, DF has been considered as an urban disease [46] with the main vector *Aedes aegypti* being common in urban areas [51,52]. *Aedes albopictus* has more recently also become abundant in urban and peri-urban areas. Further geographical expansion of *Aedes albopictus* is expected in the near future, mainly due to urbanization, migration and a changing climate [52–54].

Dengue was first reported in Vietnam in 1959 [55] and dengue has since then become endemic across the country with the circulation of all four serotypes [41,56–60]. Nevertheless, there is a spatial heterogeneity in DENV transmission in Vietnam and the incidence rate differs for each region and province. The Southern part of the country has a higher number of human cases as compared to other regions of the country [55,61]. DENV has been shown to be imported from the Southern region into the Central and Northern regions of Vietnam [62]. At present, DENV is disseminating in both urban and rural Vietnam [63,64]. However, research findings in Vietnam still pointed out that people mainly get infected with dengue when living in the cities of Hanoi (Northern part); Nha Trang, Khanh Hoa province (Central part) and Ho Chi Minh (Southern part). Less research on urban transmission has been performed in the Central Highlands although this region is also an endemic area for dengue [64].

With a warm climate and a speed of urbanization, Hanoi city has been the hotspot for dengue in the whole northern area of Vietnam and witnessed several big outbreaks during the last decade. The capital city reported 16,263 DF cases in an outbreak in 2009, which was 87% of the total number of DF cases in the whole northern region [65]. The latest as well as the largest DF outbreak so far in Hanoi occurred in 2017 with 37,651 cases and 7 deaths [66]. Four DENV serotypes (1–4) have been confirmed to co-exist in Hanoi [59]. Previous studies also revealed that the DF cases were mainly concentrated to the central urban districts [65,67–69]. In the central region of Vietnam, a recent comprehensive study in Nha Trang city revealed 12,655 dengue cases recorded between 2006 and 2016 [41]. Here it was also reported that all four serotypes of DENV were found in hospitalized patients during a single season (2015). During the peak years of DF in this coastal city, the incidence has been highest in the central urban wards [41]. The southern region of Vietnam has been known as a dengue hyperendemic area where its tropical climate constitutes favorable conditions for the breeding of the vector. Ho Chi Minh city has been identified as a critical urban center for DENV transmission, due to the high population density and intense transportations [70,71]. DENV3 was first discovered in this location of southern Vietnam and has then spread over wider areas [72]. Similar to the capital Hanoi, Ho Chi Minh city needs to cope with overloaded hospitals during the dengue outbreaks during recent years.

Vietnam is in an intense phase of industrialization and modernization leading to a rapid increase of urbanization. This factor in combination with climatic variability have influenced the distribution and density of dengue vectors [73–75]. *Aedes aegypti* is the dominant vector of DENV in urban areas of Vietnam, mainly breeding in plastic buckets, jars or flower vases [76–80]. Studies in urban areas of Hanoi city and Ho Chi Minh city identified DENV PCR-positive female *Ae. aegypti* [80,81]. *Ae. albopictus* has also been found to breed in urban and suburban areas of Vietnam, however, with lower density as compared to *Ae. aegypti*, which could be connected to climatic conditions [75,76,78,80]. Additionally, although low temperature is a negative factor for an increase of DENV vector populations, both *Ae. aegypti* and *Ae. albopictus* have been shown to survive during the winter in concrete tanks with broken lids in central districts of Hanoi city [82]. In conclusion, DENV transmission is frequently presented also in urban areas of Vietnam. Therefore, public health interventions are needed to investigate the circulation of this dangerous flavivirus since the current dengue vector control program is working inefficiently [83].

### Zika virus

ZIKV was first discovered in a rhesus monkey in Uganda, Africa in 1947 [84]. In 1954, a Nigerian female was the first human case diagnosed with a ZIKV infection [85]. Prior to 2007, ZIKV only circulated in Africa and in Asia [86,87]. However, it has since then spread to the Pacific, Europe and the Americas, with the first described ZIKV outbreak in humans on the Island of Yap – Federated States of Micronesia in 2007, where 75% of the population was infected [86,88–90]. While receiving only minor attention until 2013, ZIKV infections have now been recognized as a global threat and many countries in the Americas and in Asia had to cope with a large-scale epidemic [91]. Confronting this new situation in 2016, WHO declared ZIKV as a Public Health Emergency of International Concern [89,90]. Like DENV, ZIKV has two transmission cycles: a sylvatic cycle and a human cycle. The sylvatic transmission cycle involves non-human primates and *Aedes* mosquitoes [87]. In the human transmission cycle, ZIKV is transmitted between humans by infected *Aedes* mosquitoes [90]. In addition, ZIKV has also been isolated from other species of mosquitoes such as *Culex, Anopheles* and *Mansonia* [92,93]. However, different species of *Aedes* mosquitoes are still recognized as the main vector for human infections [86,87,92]. *Ae. aegypti* is the main vector in urban settings while *Ae. albopictus* can act as the vector of ZIKV infection in both urban and rural settings [87,92,94,95].

During recent years, new evidence revealed novel routes of transmission for ZIKV. It has been found that transfusions and perinatal transmission can transmit the virus [96,97]. Additionally, sexual contacts can also spread ZIKV among humans [98–100], which is in line with earlier indications of JEV being potentially sexually transmitted between pigs [101]. ZIKV infections usually cause milder illness with common ‘dengue-like’ symptoms such as slight fever or rash, muscle and joint pain, tiredness. The symptoms usually last from 2 to 7 days. Similarly to the other flaviviruses, the majority of the infected persons do not develop clinical symptoms [86,95]. The most severe clinical complication of ZIKV infection is congenital microcephaly of the fetus when mothers get infected during pregnancy [89,102]. A link to Guillain-Barré syndrome has also been confirmed [103,104].

In Vietnam, the attention to ZIKV has been increased during recent years. In April 2016, Vietnam declared the first Zika cases identified in two females from Nha Trang city and Ho Chi Minh city, respectively [105]. However, it should be noted that ZIKV has been retrospectively detected by RT-PCR in two children living in Ho Chi Minh city and the suburban area of the Long An province of the Southern region already in 2013 [106]. In addition, ZIKV infections have earlier been shown by the presence of neutralizing antibodies in two adults in North Vietnam in 1954 [107]. Hence, this virus appeared in Vietnam for several decades, nevertheless, because of the poor understanding of ZIKV infection and the lack of diagnostic tests at that time, it has earlier not been given any attention. After the detection of human cases in 2016, the number of recognized ZIKV infections increased dramatically.

According to statistics summarized in Table 1, a total of 212 cases of ZIKV infection were reported in 2016. This outbreak in Vietnam occurred in more than 10 cities and provinces of the Central, Central Highlands and Southern regions, mainly in urban and semi-urban areas. A number of cases were identified in pregnant women [108]; however, only one microcephaly case was found in the Dak Lak province, Central Highlands [109]. Since 2017, the ZIKV transmission decreased significantly with only 24 newly recorded cases from January to April and one newly infected province, namely Lam Dong in Central Highlands [64]. All of these cases were identified to be local mosquito-borne infections [110].

**Table 1.** ZIKV infection in Vietnam in 2016.

No	Provinces	Number of cases (212)	Region	Area
1	Ho Chi Minh City	186	Southern	Urban and semi-urban
2	Binh Duong	7	Southern	Urban
3	Binh Phuoc	1	Southern	Semi-urban
4	Can Tho	1	Southern	Rural
5	Ba Ria Vung Tau	2	Southern	Urban
6	Dong Nai	4	Southern	Urban
7	Tay Ninh	1	Southern	Semi-urban
8	Long An	1	Southern	Urban
9	Phu Yen	1	Central	Rural
10	Khanh Hoa	6	Central	Urban and semi-urban
11	Dak Lak	2	Central Highland	Rural

Source: National Institute of Hygiene and Epidemiology [64]

Vietnam is one of the five countries which have been identified as sentinel markers for ZIKV transmission [99]. Many visitors have been confirmed to have acquired ZIKV infection after their stay in Vietnam, particularly in Ho Chi Minh city, the biggest city in the country, which suffered the most severe outbreak. Israel and Korean travelers were suspected to have been infected with ZIKV in Vietnam in early 2016, while Zika disease was not reported at all in Vietnam in 2015 [111,112]. After that, two cases, a German woman and a Japanese man were hospitalized in Japan in September and November 2016, respectively, and were both diagnosed to be infected by ZIKV in Ho Chi Minh city [113,114]. Taiwan Centers for Disease Control recorded five ZIKV cases originating from Vietnam in 2016, and one new case was confirmed in a person who worked in Vietnam in late 2018 [115]. Therefore, it seems that ZIKV is still circulating in Vietnam.

Although unsafe sexual practices and blood transfusions can be a route for ZIKV transmission, no such cases have been identified in Vietnam. The most important risk factor of ZIKV transmission is referred to the high density of the main vector, the *Aedes* mosquito population [110]. In an entomological report from Nha Trang city in 2016, ZIKV existed in *Ae. aegypti* with a high prevalence of 0.24% [116]. This was in accordance with an entomological finding in an urban district of Brazil where three out of one hundred and ninety-eight pools of *Ae. aegypti* were naturally infected with ZIKV [117]. In Vietnam, the practice of storing water in tanks or containers at home is common, enabling *Aedes* mosquitos, the main vector of DENV and ZIKV, to expand quickly [110]. In addition, the impact of climate change and urbanization plus the lack of awareness among urban inhabitants on the importance of removing the breeding grounds of mosquitoes have lead to a significant increase of the vector density [33,83]. In addition to encouraging people to use personal protective measures to avoid mosquito bites, new effective strategies for vector control need to be built globally [118] because the current program has its limitations and is difficult to achieve [119]. Hence, ZIKV is concentrated mainly in urban and semi-urban areas of the Central, Central Highlands and Southern regions of Vietnam, but there is a significant risk that this virus can re-emerge in the Northern region due to a huge human migrations [110].

### Other flaviviruses

Quang Binh virus is another flavivirus that was isolated from *Cx. tritaeniorhynchus* mosquitoes collected in the Quang Binh province, Central Vietnam, in 2002 [120]. However, no human infection has yet been detected, and no circulation in mosquitoes has been shown since 2002. Knowledge of the epidemiology, particularly regarding urban transmission, of this virus in Vietnam is still limited.

Some important flaviviruses have never been detected in Vietnam. YFV shares the same mosquito vector as DENV and ZIKV (*Ae. aegypti)* and a similar urban cycle of viral transmission. However, interestingly, YFV has never been identified in Asia, so far only in Africa and the Americas [121,122].

WNV is distributed extensively in Africa, the Americas, parts of southern Europe, the Middle East and western Asia. Viral transmission to humans has mainly been associated with bites by *Culex* mosquitoes. Eighty percent of the infections are asymptomatic or mild febrile illness. However, 1 in 150 WNV infections results in meningitis or encephalitis, and even deaths [2,123]. It is not yet known why Southeast Asia has so far been spared from this flavivirus, and more research into the vector potential of the regional *Culex* species may be warranted.

## Conclusion

This review focuses on flaviviruses in urban Vietnam, in order to bring more clarity to its transmission in cities (see in the table 2). In conclusion, three of the most important flaviviruses: JEV, DENV and ZIKV co-exist in urban Vietnam and pose a major risk for severe diseases in humans via mosquito bites.

**Table 2.** Summary points of urban transmission of mosquito-borne flavivirus in Vietnam.

Flavivirus	Vector	Amplifying host in cities	Risks in the cities	Summary
Japanese encephalitis virus	The major vector *Culex tritaeniorhynchus* prefers breeding in rice paddies but can be found in cities. Other vectors, such as *Cx. quinquefasciatus* can breed in sewers in the urban areas	Pigs present in urban agriculture or transported for slaughter	JEV is circulating with low risk in cities	JEV has potential for transmission in urban areas mainly whereurban livestock keeping is presented.
Dengue virus	Both *Aedes aegypti* (principal vector) and *Aedes albopictus* (competent vector) breeds easily in cities	Humans	Dengue is hyperendemic from 1960s in large Vietnamese cities	Dengue is circulating all year round with all four serotypes but higher risk in the warmest season. Outbreaks mainly occur in urban areas across the country.
Zika virus	Sameas dengue	Humans	ZIKV is re-emerging as an outbreak in 2016 – early 2017, and but fewer case have been confirmed afterwards	ZIKV has been more common in cities and peri-urban areas of central, central highlands and southern region.
Quang Binh virus	Limited information on epidemiology			
Yellow Fever virus	Not found but could potentially be introduced in urban Vietnam			
West Nile virus	Not found but could potentially be introduced in urban Vietnam			

The urban epidemiology of JEV has mainly been studied in three regions of Vietnam. Several potential mosquito vectors for JEV transmission have been identified in entomological surveys; found RT-PCR positive for this virus. JEV has the potential to rapidly emerge in urban communities, particularly when pig-keeping is practiced in peri-urban and urban areas of cities, as frequently occurring in Vietnam. DENV is the most widespread flavivirus in Vietnam where all DENV serotypes are circulating. They are regularly transmitted in urban and semi-urban areas yearly throughout the country and constitute a major threat; mainly in the larger cities. ZIKV was first detected in the Northern region of Vietnam in the 1950s but seems to have recently re-emerged only in urban areas of the Central, Central Highlands and Southern regions, especially in Ho Chi Minh city, where migration and international travel are contributing to risks of further spread. In conclusion, there is an urgent need for intensified research to be able to create new appropriate strategies to cope with the increasing mosquito populations during the high-risk season and to prevent human infections from the flaviviruses.
